# The Outcomes of Enamel Matrix Derivative on Periodontal Regeneration under Diabetic Conditions

**DOI:** 10.3390/medicina57101071

**Published:** 2021-10-08

**Authors:** Laura Elena Narita, Alexandru Mester, Florin Onisor, Simion Bran, Maria Ioana Onicas, Andrada Voina-Tonea

**Affiliations:** 1 Faculty of Dental Medicine, University of Medicine and Pharmacy „Iuliu Hatieganu”, 400012 Cluj-Napoca, Romania; laura.elen.narita@elearn.umfcluj.ro (L.E.N.); maria.ioan.onicas@elearn.umfcluj.ro (M.I.O.); 2 Department of Oral Health, University of Medicine and Pharmacy „Iuliu Hatieganu”, 400012 Cluj-Napoca, Romania; 3 Department of Maxillofacial Surgery and Implantology, University of Medicine and Pharmacy „Iuliu Hatieganu”, 400012 Cluj-Napoca, Romania; dr_brans@umfcluj.ro; 4 Department of Dental Materials, University of Medicine and Pharmacy „Iuliu Hațieganu”, 400012 Cluj-Napoca, Romania; andrada.tonea@umfcluj.ro

**Keywords:** enamel matrix proteins, EMD, diabetes, tissue repair, periodontal regeneration

## Abstract

*Background and Objectives*: Enamel matrix derivative (EMD) is a biomaterial used for periodontal regenerative therapy due to its properties of stimulating cementum development and bone synthesis. Diabetes is a chronic condition that affects healing and predisposes to infection. The aim of this review was to evaluate the current studies available on the application and results of EMD for periodontal regenerative therapy under diabetic conditions. *Materials and Methods*: Five databases (PubMed, ResearchGate, Scopus, Web of Science and Google Scholar) were searched for relevant articles, using specific keywords in different combinations. The inclusion criteria were clinical trials, case reports, case studies, and animal studies published in English, where periodontal treatment for bone defects includes EMD, and it is performed under diabetic conditions. *Results*: Of the 310 articles resulted in search, five studies published between 2012 and 2020 met the inclusion criteria and were selected for the current review. In human studies, the use of EMD in infrabony defects showed favorable results at follow-up. In animal studies, periodontal regeneration was reduced in diabetic rats. *Conclusions*: EMD might promote bone healing when used under diabetic conditions for the regenerative periodontal therapy. Due to limited number of studies, more data are required to sustain the effects of EMD therapy in diabetic settings.

## 1. Introduction

Currently, diabetes represents a worldwide challenge, for both patients and medical professionals [1]. Diabetes mellitus is a complex syndrome, induced by the disturbance of insulin secretion or by the resistance of the peripheral cells to the action of insulin [1]. Type 1 diabetes is characterized by the destruction of pancreatic β cells, leading to a complete insulin deficiency, while type 2 is expressed by insulin resistance and relative insulin deficit [1]. The most common consequence in both types is hyperglycemia, characterized by the increase in glucose levels in blood circulation, above the optimal limits. According to the WHO, more than 400 million individuals all over the globe are impacted by diabetes and almost 2 million deaths caused by the disease have been reported at this point [2].

The presence of diabetes mellitus in dental patients is of great importance, given the close interdependence between diabetes and periodontal disease [1,2]. Periodontitis is characterized by an initial microbial aggregation in the supra and subgingival regions, leading to inflammation processes and destruction of the periodontal tissues [3]. Studies have shown that the possibility of developing periodontitis is up to three times higher for persons who suffer from diabetes, while patients with periodontal disease have higher risks of diabetes occurrence [4]. Furthermore, the lack of regulation in glycemic blood levels increases the inflammatory feedback of the organism, including the periodontal tissue [5]. Contrarily, therapeutic approaches of periodontal pathologies can improve the management of glycemia [6]. Regarding the periodontal treatment, research has shown that mechanical therapeutic measurements but also medication concur to the amelioration of inflammation and glycemia levels [7]. Particular attention should be given to the link between periodontal disease and microangiopathy, which was proven to exist in the case of type 2 diabetes [8]. It has been determined that negative consequences in the structure of the periodontium can occur due to diabetes type 2 microangiopathy [8,9]. 

In 2017, the International Diabetes Federation and the European Federation of Periodontology have published a consensus paper [3] regarding the approach of periodontal disease in patients with diabetes. Proper oral health care instructions given by the dentist are essential [3]. Twice a day teeth cleaning using specific antiplaque toothpaste and mouthwash and the use of interdental brushes or floss are highly recommended for this type of patient [3,4]. Due to the high risk of developing cavities, in case of massive tooth loss, complex oral rehabilitation is needed, in order to restore the masticatory function and also to ensure proper nutrition [3,5]. Informing diabetic patients about the effects of periodontal disease on the metabolic system is crucial, due to their direct cause–effect relationship. When diagnosed, periodontitis should be treated immediately and then a preventive routine and regular periodontal supervision are elemental. If periodontitis is not treated, in addition to uncontrolled diabetic parameters there is a risk of cardiovascular and renal system issues [3,4,5,6]. Regarding periodontal surgical therapy, it can be performed on patients with well-controlled diabetic parameters, with similar results to those obtained in healthy patients. Special attention is required when the patient scheduled for periodontal surgery is treated with insulin or sulfonylureas, a consult with the physician regarding the time of the surgery and also the dosage of the drugs is vital for the prevention of hypoglycemia during the procedure [3,4,5,6]. Alongside periodontitis, other potential complications such as dental cavities caused by a dry mouth, a sensation of burning mouth and candida infections may occur. In case of bleeding gums while brushing or eating, halitosis, teeth mobility, gingival recessions with black triangles, or a gingival abscess, an annual periodontal check-up is suggested [3,4,5,6].

After non-surgical periodontal therapy, periodontal structures may need to be repaired using different types of regenerative products. One of these products that can have regenerative outcome is enamel matrix derivate (EMD). EMD is a protein aggregate extracted from the porcine fetal teeth that are thought to mediate periodontal regeneration by miming the process of tooth formation [10]. Even though EMD is a material derived from porcine teeth, no immune reactions during periodontal treatment were described in the literature. It contains up to 90–95% amelogenins [10,11], which participate in the enamel and periodontal attachment formation during odontogenesis [12]. Under certain physiological circumstances, the proteins assemble themselves and form an extracellular matrix, which inside the human body is digested slowly by matrix metalloproteinases [10]. As the process unfolds, weeks after EMD application, bioactive peptides are released around the periodontal defect [10]. EMD contains as well enamelin and ameloblastin [13], the last one being involved in the inhibition of epithelial cell proliferation [14]. Furthermore, the vehicle solution, PGA, has proved to have antimicrobial properties on the bacteria associated with periodontal disease [15]. Other modifications induced by EMD are represented by increased production of PGE2 and OPG, proliferation and migration of T-lymphocytes, and a decreased production of IL-1b, IL-8 [11]. Besides influencing wound healing by downregulating inflammatory genes, while upregulating growth and repair-promoting genes, EMD has been demonstrated to encourage angiogenesis by the activation of endothelial cells [16,17,18,19], and it also stimulates microvascular cell differentiation [11]. There is evidence that EMD exerts a significant influence on the behavior of periodontal ligament cells, osteoblasts, cementoblasts, gene expression by regulating cell attachment, proliferation, and differentiation [13]. All these actions promote EMD as being a promising antagonist to the most clinical outcomes of diabetes, such as: general inflammation, periodontal tissue destruction, vascular dysfunction, immune system suppression, alteration of bone metabolism [20,21,22]. There is already solid evidence of the clinical efficacy of EMD on patients without systemic conditions. Considering the ethical aspects for commencing clinical trials, the limited studies available on individuals with diabetes mellitus are not enough to determine the exact outcomes of EMD; although, it is thought that this product may ameliorate or even neutralize the effects of diabetes and thus enhance the healing process [22]. Having the cellular and chemical changes under application of EMD mentioned above, on a macro level, they are translated into clinical attachment gain, reduction of gingival recession and of the pocket depth. The main mechanisms suspected for wound healing are both angiogenesis and anti-inflammatory actions [20,22].

Enamel matrix derivate found its application in periodontology, being used for regenerative procedures, in order to stimulate wound healing, cementum and bone formation [17,18]. So far, EMD has been used in patients without systemic disease, showing great results. In regard to this, the aim of this review was to evaluate what outcomes can be achieved by using EMD for periodontal regenerative procedures under diabetic conditions.

## 2. Materials and Methods

The purpose of this section is to detail our systematic approach following the guidelines of the Preferred Reporting Items for Systematic Review and Meta-Analysis (PRISMA) [19]. The research question was: “Is EMD effective in periodontal regenerative procedures under diabetic conditions?”

### 2.1. Eligibility Criteria

The inclusion criteria consist of clinical trials, case studies, case reports, or animal studies where periodontal defects were treated with EMD under diabetic conditions. The exclusion criteria were the following: letters to editors, unpublished or incomplete data, and conference papers.

### 2.2. Literature Search

PubMed, ResearchGate, Scopus, Web of Science and Google Scholar databases were searched to find all relevant studies published in English from the date of inception up to April 2021. A combination of keywords was used, as follows: (“enamel matrix proteins” OR “EMD” OR “Emdogain”) AND (“diabetes” OR “diabetes mellitus” OR “diabetic patients”). Titles and abstracts of the articles identified on the databases searched were assessed for eligibility and irrelevant articles were excluded. Full-text articles previously obtained were read and assessed in order to correspond to the inclusion criteria. The lists of references of the studies included were also searched. 

### 2.3. Data Extraction and Statistical Analysis

The following data from the human studies were extracted using a standard data collection form: first author, year of study, country, type of study, characteristics of the patients, mean age, periodontal measurements, results before and after the treatment and conclusions. As for animal studies, the following data were extracted: first author, year of study, country, type of study, animal type, measurements, results, and conclusions. Due to heterogeneity of the included studies, an assessment of risk of bias and a statistical analysis could not be achieved.

## 3. Results

### 3.1. Search Results and General Characteristics

**Figure 1:**
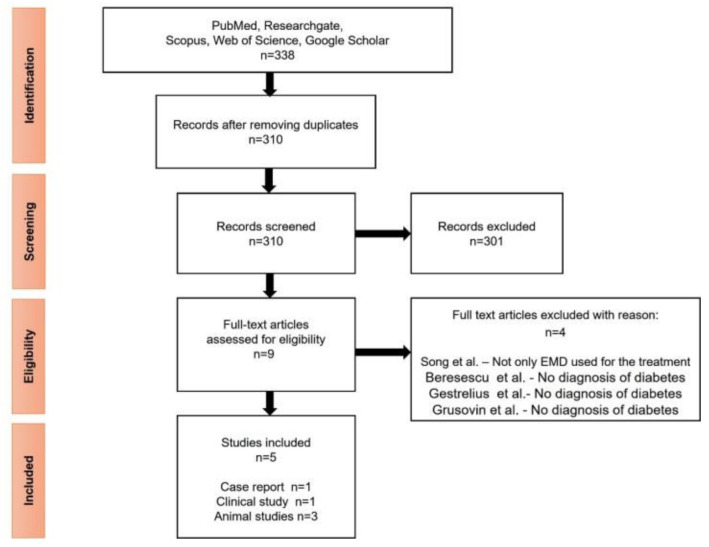
Flow chart of methodology according to PRISMA.

A total of 338 studies were provided from Medline (through PubMed), ResearchGate, Scopus, Web of Science and Google Scholar databases. After removing the duplicates, a total of 310 articles were screened. The articles were reviewed by title and abstract, of which 9 articles were identified for full-text assessment. Out of these, 5 articles met the inclusion criteria at the end of the analysis [20,21,22,23,24]. Reasons for the exclusion of reviewed full-text articles [25,26,27,28] are shown in Figure 1. The 5 studies meeting the inclusion criteria were published between 2012 and 2020. Of these, one article is a prospective clinical study [22], one case report [24], and three studies are on rats [20,21,23]. The studies were conducted in 3 countries: Japan [20,22,24], Switzerland [21], and Brazil [23].

### 3.2. Periodontal Disease Assessment and Surgical Intervention

Mizutani and collaborators [22] investigated the regenerative outcomes of minimally invasive surgical technique (MIST) or modified MIST with EMD in infrabony defects in patients with type 2 DM. The included cohort had chronic periodontitis and was evaluated regarding O’Leary full-mouth plaque control record (PCR), tooth mobility, periodontal probing depth (PPD), clinical attachment loss (CAL), gingival recession (REC), bleeding on probe (BOP), and intra-oral radiographs. Seshima and collaborators [24] reported in their case report a patient with generalized chronic periodontitis and type 2 DM. Periodontal disease was assessed according to PPD, BOP, tooth mobility, the implication of the furcation and radiographic examination. In order to evaluate the oral hygiene, the O’Leary full-mouth plaque control record was used and the assessment of the oral health-related quality of life (OHRQL) was carried out through a questionnaire.

Takeda and coworkers [20] investigated the effects of EMD in diabetic Wistar rats induced with streptozocin. In the maxilla molar region, three-wall intrabony defects were created, bilaterally, then EMD or saline solution was applied. After 28 days, the maxilla was harvested and assessed through histomorphometrically analysis which consists of the following measurements: cement–enamel junction to the bottom of the bone defect, junctional epithelium length, area of the new bone and cementum. The recently mineralized tissue formed was detected with micro-CT analysis. Additionally, immediately after the surgery and 1 week post-op, intraoral digital images were taken in order to evaluate the closure of the wound. In another study, Shirakata and collaborators [21] aimed to evaluate the short-term effects of EMD in supra and/or infrabony defects in diabetic rats. On the mesial root of the first maxillary molars, periodontal defects were created surgically, followed by the application of EMD on only one side of the maxilla. After 3 weeks, biopsy blocks were taken from rats’ maxilla and were histomorphometrically analyzed. The measurements included the root length, defect depth, sulcus depth, gingival recession, length of junctional epithelium, supracrestal connective tissue, area of newly formed bone and cementum, presence of ankylosis. Corrêa et al. [23] performed also a histomorphometrically analysis to evaluate the effect of EMD in periodontal defects in diabetic rats. Bone defects were created surgically on the buccal aspect of the first mandibular molar. After 3 weeks, rats were euthanatized and bone blocks were analyzed to assess defect fill, density of newly formed bone, new cementum formation and the number of osteoclasts. 

### 3.3. The Outcome of EMD under Diabetic Condition

In the prospective study conducted by Mizutani [22], the results for intrabony defects were favorable at the follow-up; clinical attachment was gained (3.8 ± 1.1 mm at 3 years), and the radiographic examination showed bone formation (58.3 ± 10.4% at 3 years) (Table 1). The authors mentioned that the use of MIST/M-MIST approach was a useful technique in DM patients because is considered to be less invasive and more predictable. This technique offers high success rates due to a minimal surgical invasion which determines a blood flow recovery, accelerated healing and tissue repair. Another advantage is the small amounts of analgesics that patients need to take postoperatively. 

In Seshima’s case report [24], it has been observed that periodontal parameters improved, the clinical attachment was gained (from 1 to 4 mm), and the probing depths were reduced (reduction of ≥4 mm) at 7 months (Table 1). In addition to the periodontal parameters, the diabetic condition did also improve after the treatment, with a decrease remarked in the glycated hemoglobin and the fasting plasma glucose. The authors have mentioned that the results were attributed due to improvement of glycated hemoglobin values following initial non-surgical periodontal therapy, which was performed in close collaboration with the physician.

The animal studies [20,21,23] showed bone regeneration in all rats, although it was reduced in diabetic rats compared to non-induced diabetes rats (Table 2). Further, it was observed that the formation of the cementum in diabetic rats was not meaningful. Takeda [20] demonstrated that EMD is able to promote wound healing and tissue regeneration in diabetic rats. The results obtained in histological analysis were similar to micro-CT analysis. The author has stated that low bone regeneration was observed also in the low levels of angiogenic and osteogenic markers. Tissue regeneration in DM rats may have come from EMD which regulates the angiogenic factors. In the end, the author concluded that EMD may have a beneficial effect in diabetic settings. Shirakata [21] observed that the limit between old and new bone. New bone formation was localized in the apical region of infrabony defect being considered to be a result of an early phase of healing and repair after surgical procedure. On the other hand, histological assessment showed that EMD did not influence bone healing. Another important aspect was the presence of a more gingival recession in diabetic rats compared to healthy rats; this may be due to an increased lack of collagen synthesis and increased degradation and solubility of gingival tissues. In this research, the author concluded that EMD had no influence on bone healing during short-term healing in diabetic rats. Corrêa et al. [23] noticed that defect fill and bone density were lower in diabetic rats; this can be explained by the low appositional rate of bone formation which EMD is not able to enhance bone density. Additionally, EMD was not able to promote new cementum formation. On TRAP staining, authors have stated that EMD can stimulate osteoclasts and osteoblasts in order to provide an environment favorable for bone formation. In conclusion, the authors of this paper mentioned that EMD may be able to fill a bone defect but is not able to increase bone density and formation of new cementum in diabetic rats.

## 4. Discussion

Given the fact that the oral cavity provides a permanent microbial community and can act as a wellspring for their pathological dissemination, the screening of periodontal disease in diabetic patients should be a part of the routine prophylactic approaches, while special care should be offered to diabetic patients with periodontal disease [1,2,3,4]. Since both diabetes and periodontitis need a lifelong treatment strategy, compliance from the patient is essential [4,29]. When diagnosed, the periodontal treatment should be initiated without delay, in order to reduce the subgingival bacterial load and, consequently, the circulating bacteria and its products [3]. The consensus paper written in 2017, at the World Workshop by Tonnetti and coworkers, proposed a new framework for periodontal disease according to staging and grading system; staging consists of the severity and the complexity of disease management and grading offers further data about biological features of the disease [30]. A novelty introduced in the grading part consists of adding risk factors such as smoking and diabetes, which are grade modifiers. The value of HbA1c levels according to the progression of periodontal disease consists of grade A (normoglycemic patient or no diabetes, with slow rate of periodontitis progression), grade B (diabetes patient with HbA1c <7%, moderate rate of periodontitis progression) and grade C (diabetes patient with HbA1c >7%, rapid rate of periodontitis progression) [30]. When choosing a surgical or non-surgical periodontal therapy in these patients, blood glucose and HbA1c levels should always be taken.

Enamel matrix derivative promotes periodontal tissue regeneration by various cell modulation effects such as the suppression of inflammation and the promotion of angiogenesis. As a result, it is suggested that using EMD for the regenerative periodontal therapy decreases the negative effect of diabetes on wound healing and periodontal tissue regeneration [22]. Koji Mizutani et al. [22], have achieved significant clinical attachment gain and radiographical bone fill in the DM group at a level comparable with the non-DM group, based on a 3-year observation. Subgroup analysis in the DM group showed no significant difference in the distribution of well or poorly controlled DM and CAL gain, nor significant difference for age. Their reports show a positive result of EMD treatment from the very beginning, by obtaining full primary flap closure, which is essential for wound healing. An interesting observation is that similar results were obtained in elderly individuals, when compared to the younger subjects. One explanation would be the angiogenetic effect of EMD, which helped to enhance metabolism in the area. In Seshima et al.’s [24] case report, significant results were obtained in both CAL and PD measurements, 7 months after the regenerative periodontal treatment. The overall periodontium status was improved, even if during supportive periodontal therapy the patient’s HbA1c levels increased under unknown cause. In this study, the periodontal therapy was implemented only after HbA1c levels dropped under the threshold level, which is 6.9%, according to the Japanese Society of Periodontology. Nevertheless, EMD can be stressful for the root surface, during the resorption process of the gel matrix an inflammatory process can be activated due to the degradation with the activation of MMP8 and the resorption of the root surface or more complicated ankylosis [31].

As regards the effect of EMD demonstrated on diabetic rats, the length of the junctional epithelium was significantly shorter in EMD-applied sites in Shirakata et al.’s article [21] The results of the study could not confirm that EMD enhances wound healing and connective tissue regeneration. In the study conducted by Kohei Takeda et al. [20], the regenerative effect of EMD was diminished on diabetic rats, still, it was noticed even in rats with uncontrolled DM. Additionally, they reported that the primary wound closure was decent one week after surgery in general, but EMD treated sites had higher rates, which can be translated as the result of enamel matrix derivative’s direct effect on the tissue. Interestingly, with regard to wound healing and newly formed bone, they reported that the diabetic animals group treated with EMD had similar results with the controlled group without application of EMD, which led us to the assumption that EMD reduced the diabetic conditions. A measurable layer of new cementum was not detected in any of the teeth in the study, EMD had no observable positive effect on cementum formation in both diabetic and non-diabetic animals. Furthermore, the results on EMD therapy were not very promising for the diabetic group in Corrêa et al.’s study [23]. Neither bone density nor new cementum formation was observed in the DM group. One noticeable difference before and after was the substantial defect fill with EMD in both categories (DM and non-DM rats). As seen in the included studies of this review, EMD may not offer the appropriate results for infrabony defects in diabetic settings. Diabetes affects bone metabolism by enhancing osteoclastogenesis and increasing apoptosis among osteoblasts. Therefore, patients with diabetes have a higher risk of bone fractures due to reduced bone density or due to decreased bone quality. The bone turnover rate is reduced. Enhanced osteoclastogenesis is present because of the chronic inflammation throughout the body, as well as at the periodontium level, increasing the risk of developing periodontal disease for diabetic patients [21]. Therefore, alternatives such as platelet-rich fibrin with/without inorganic bovine bone or autologous bone alone or combined with xenogenic bone should be taken into account [32,33].

The strengths of our review was the systematic search into five database. Additionally, our review is the first to gather information from both human and animal studies in regard to the effect of EMD in diabetic settings. Of course, our research has several limitations. One of them is the small number of human patients with diabetes mellitus treated using EMD. Further, the diagnosis of periodontal disease and the risk factors for it was not detailed in the studies. Another drawback is represented by the assessment of the periodontal parameters, which was not explicit and, as a result, the reproducibility of the measurements may not be accurate. Additionally, the follow-up period after the periodontal regenerative therapy was not detailed. In addition, the results obtained in humans and animals were slightly different.

## 5. Conclusions

Enamel matrix derivate might promote bone healing when used under diabetic conditions for the regenerative periodontal therapy. In diabetic rats, bone regeneration was obtained after the periodontal treatment, but it was diminished compared to the non-induced diabetic rats’ group. As for humans, the improvement of the periodontal parameters was noticed, and it was similar between diabetic and non-diabetic patients. More data are necessary to sustain the effects of enamel matrix derivate in diabetic patients.

## Figures and Tables

**Table 1:** Human studies.

Studies Characteristics				Periodontal Disease Assessments			
Author. Year. Country.	Study Type	Sample Size	Characteristics	Measurements	Results		Conclusions
Mizutani, 2020, Japan [22]	Prospective cohort study	DM group: n = 10 Non-DM group: n = 18	DM group: (a) Mean age: 67.5 ± 7.6 (b) Sex: male (n = 4) female (n = 6) (c) BMI: 23.9 ± 3.1 d) HbA1c: 6.82 ± 0.72% Non-DM group: (a) Mean age: 63.1 ± 9.7 (b) Sex: male (n = 5) female (n = 13) (c) BMI: 22.8 ± 2.7 (d) HbA1c: NA	PPD CAL PCR REC BOP Periapical radiographs Tooth mobility	**Intrabony Defects**		The combination of minimally invasive surgical technique and EMD for the regenerative periodontal surgery showed successful results for both DM and non-DM groups.
					**Pre-operative** **PPD (mm)** (a) DM group 7.1 ± 1.6 (b) Non-DM group 7.0 ± 1.3 **CAL (mm)** (a) DM group 7.6 ± 1.5 (b) Non-DM group 7.8 ± 1.5 **Radiographical defect (mm)** (a) DM group 4.6 ± 0.9 (b) Non-DM group 4.8 ± 1.5	**Post-operative (1 year and 3 years)** **PPD (mm)** (a) DM group 4.8 ± 1.5; 4.5 ± 1.4 b) Non-DM group 4.8 ± 1.5; 4.7 ± 1.4 **CAL** (a) DM group 4.1 ± 1.2; 3.8 ± 1.1 (b) Non-DM group 4.3 ± 1.1; 4.1 ± 1.1 **Radiographical defect (mm)** (a) DM group 2.5 ± 0.7; 2.6 ± 0.6 (b) Non-DM group 3.1 ± 1.4; 3.2 ± 1.3	
Seshima, 2016, Japan [24]	Case report	n = 1	Age: 66 years Gender: male HbA1c: 7.8%	CAL PPD Furcation Tooth mobility OHRQL	**Intrabony Defects**		The use of EMD in intrabony defects had favorable outcomes.
					**Pre-operative** **PPD (mm)** Tooth 1.6: 10 Tooth 2.6: 7 Tooth 2.7: 9 **CAL (mm)** Tooth 1.6: 10 Tooth 2.6: 7 Tooth 2.7: 9	**Post-operative (7 months)** **PPD (mm)** Tooth 1.6: 5 Tooth 2.6: 3 Tooth 2.7: 4 **CAL (mm)** Tooth 1.6: 6 Tooth 2.6: 4 Tooth 2.7: 8	

CAL: clinical attachment loss; PPD: periodontal probing depth.

**Table 2:** Animal studies.

Author. Year. Country.	Sample Size, Type of Animal	Measurements	Results	Conclusions
Takeda, 2018, Japan [20]	DM group: n = 18 Control group: n = 18 Male Wistar rats	Histomorphometrically analysis: cement–enamel junction to the bottom of the bone defect, length of the junctional epithelium, newly formed cementum, new bone, area of new bone, and area of new cementum. Micro-CT analysis: bone volume, cancellous bone volume, bone mineral density, cancellous mineral content	Defects were filled with new connective tissue, new cementum, and new bone after 28 days in both groups. In the DM group, sparse and oblique collagen fibers were detected; in the control group, dense and vertical collagen fibers were present. Newly formed connective tissue attachment and bone were increased at EMD-treated sites.	Bone regeneration was noticed when EMD was used, but it was diminished in DM rats.
Shirakata, 2014, Switzerland [21]	DM group: n = 15 Control group: n = 15 Male Wistar rats	Histomorphometrically analysis: root length, defect depth, sulcus depth, gingival recession, length of junctional epithelium, supracrestal connective tissue, new bone, new cementum, ankylosis	Root length and defect depth values were comparable in all groups and there were no statistical difference. Gingival recession was greater in diabetic rats. The length of junctional epithelium was smaller in the EMD-treated sites of both DM and control animals. Sulcus depth and length supracrestal connective tissue differences were not seen between the groups. New bone formation was mostly situated on the apical site of the defect. New cementum has been seen in none of the groups.	EMD had no benefits in the formation of new bone and cementum.
Corrêa, 2012, Brazil [23]	DM: n = 10 Non-DM: n = 10 Male Wistar rats	Defect fill The density of newly formed bone New cementum formation Number of osteoclasts	Defect fill: DM showed less defect fill than control, for the EMD-treated defects and non-treated controls. Bone density: Statistically significant differences were noticed in bone density between DM and non-DM, in the EMD-treated sites and the non-treated controls. New cementum formation: No statistically significant difference in new cementum formation between DM and non-DM, for EMD-treated sites and non-treated control.	EMD determined an increased defect fill in both groups and enhanced bone density and new cementum formation only in non-diabetic animals.

## Data Availability

Not applicable.
